# Supplementary material for: Unveiling the Bioactive Potential of the Invasive Jellyfish Phyllorhiza punctata Through Integrative Transcriptomic and Proteomic Analyses
Source: Biomolecules. 2025 Aug 4;15(8):1121. doi: 10.3390/biom15081121 (PMC12383608; doi:10.3390/biom15081121)
Supplement: Supplementary file 1 [file biomolecules-15-01121-s001.zip › Suplementary Material/FileS3/PEPFOLD4/XP_065069500_15627/clusters.html]

 PEP-FOLD clusters


Download archive of all models  here   

|  |  |  |
| --- | --- | --- |
| # | file | sOPEP |
| # cluster 1 - Size: 1 |  |  |
|  | XP\_065069500\_15627-model1 | -13.6885 |
| # cluster 2 - Size: 3 |  |  |
|  | XP\_065069500\_15627-model2 | -11.3832 |
|  | XP\_065069500\_15627-model2.1 | -11.3832 |
|  | XP\_065069500\_15627-model2.2 | -11.3091 |
| # cluster 3 - Size: 10 |  |  |
|  | XP\_065069500\_15627-model3 | -10.0275 |
|  | XP\_065069500\_15627-model3.1 | -9.89268 |
|  | XP\_065069500\_15627-model3.2 | -9.78232 |
|  | XP\_065069500\_15627-model3.3 | -9.78232 |
|  | XP\_065069500\_15627-model3.4 | -9.73702 |
|  | XP\_065069500\_15627-model3.5 | -9.7221 |
|  | XP\_065069500\_15627-model3.6 | -9.63549 |
|  | XP\_065069500\_15627-model3.7 | -9.55405 |
|  | XP\_065069500\_15627-model3.8 | -9.54826 |
|  | XP\_065069500\_15627-model3.9 | -9.32232 |
| # cluster 4 - Size: 12 |  |  |
|  | XP\_065069500\_15627-model4 | -9.68604 |
|  | XP\_065069500\_15627-model4.1 | -9.52438 |
|  | XP\_065069500\_15627-model4.2 | -9.52262 |
|  | XP\_065069500\_15627-model4.3 | -9.42057 |
|  | XP\_065069500\_15627-model4.4 | -9.42057 |
|  | XP\_065069500\_15627-model4.5 | -9.42057 |
|  | XP\_065069500\_15627-model4.6 | -9.42057 |
|  | XP\_065069500\_15627-model4.7 | -9.37115 |
|  | XP\_065069500\_15627-model4.8 | -9.33246 |
|  | XP\_065069500\_15627-model4.9 | -9.33246 |
|  | XP\_065069500\_15627-model4.10 | -9.33246 |
|  | XP\_065069500\_15627-model4.11 | -9.33047 |
| # cluster 5 - Size: 74 |  |  |
|  | XP\_065069500\_15627-model5 | -9.52 |
|  | XP\_065069500\_15627-model5.1 | -9.46775 |
|  | XP\_065069500\_15627-model5.2 | -9.46378 |
|  | XP\_065069500\_15627-model5.3 | -9.436 |
|  | XP\_065069500\_15627-model5.4 | -9.436 |
|  | XP\_065069500\_15627-model5.5 | -9.436 |
|  | XP\_065069500\_15627-model5.6 | -9.436 |
|  | XP\_065069500\_15627-model5.7 | -9.436 |
|  | XP\_065069500\_15627-model5.8 | -9.436 |
|  | XP\_065069500\_15627-model5.9 | -9.436 |
|  | XP\_065069500\_15627-model5.10 | -9.436 |
|  | XP\_065069500\_15627-model5.11 | -9.436 |
|  | XP\_065069500\_15627-model5.12 | -9.436 |
|  | XP\_065069500\_15627-model5.13 | -9.436 |
|  | XP\_065069500\_15627-model5.14 | -9.436 |
|  | XP\_065069500\_15627-model5.15 | -9.436 |
|  | XP\_065069500\_15627-model5.16 | -9.436 |
|  | XP\_065069500\_15627-model5.17 | -9.436 |
|  | XP\_065069500\_15627-model5.18 | -9.436 |
|  | XP\_065069500\_15627-model5.19 | -9.436 |
|  | XP\_065069500\_15627-model5.20 | -9.436 |
|  | XP\_065069500\_15627-model5.21 | -9.436 |
|  | XP\_065069500\_15627-model5.22 | -9.436 |
|  | XP\_065069500\_15627-model5.23 | -9.436 |
|  | XP\_065069500\_15627-model5.24 | -9.436 |
|  | XP\_065069500\_15627-model5.25 | -9.436 |
|  | XP\_065069500\_15627-model5.26 | -9.41491 |
|  | XP\_065069500\_15627-model5.27 | -9.40513 |
|  | XP\_065069500\_15627-model5.28 | -9.39933 |
|  | XP\_065069500\_15627-model5.29 | -9.38806 |
|  | XP\_065069500\_15627-model5.30 | -9.38806 |
|  | XP\_065069500\_15627-model5.31 | -9.38806 |
|  | XP\_065069500\_15627-model5.32 | -9.38806 |
|  | XP\_065069500\_15627-model5.33 | -9.38806 |
|  | XP\_065069500\_15627-model5.34 | -9.38806 |
|  | XP\_065069500\_15627-model5.35 | -9.38806 |
|  | XP\_065069500\_15627-model5.36 | -9.38806 |
|  | XP\_065069500\_15627-model5.37 | -9.38806 |
|  | XP\_065069500\_15627-model5.38 | -9.38806 |
|  | XP\_065069500\_15627-model5.39 | -9.37851 |
|  | XP\_065069500\_15627-model5.40 | -9.35922 |
|  | XP\_065069500\_15627-model5.41 | -9.33613 |
|  | XP\_065069500\_15627-model5.42 | -9.33613 |
|  | XP\_065069500\_15627-model5.43 | -9.33613 |
|  | XP\_065069500\_15627-model5.44 | -9.33613 |
|  | XP\_065069500\_15627-model5.45 | -9.33613 |
|  | XP\_065069500\_15627-model5.46 | -9.33613 |
|  | XP\_065069500\_15627-model5.47 | -9.33613 |
|  | XP\_065069500\_15627-model5.48 | -9.33613 |
|  | XP\_065069500\_15627-model5.49 | -9.33613 |
|  | XP\_065069500\_15627-model5.50 | -9.32553 |
|  | XP\_065069500\_15627-model5.51 | -9.32553 |
|  | XP\_065069500\_15627-model5.52 | -9.32553 |
|  | XP\_065069500\_15627-model5.53 | -9.32553 |
|  | XP\_065069500\_15627-model5.54 | -9.32553 |
|  | XP\_065069500\_15627-model5.55 | -9.32553 |
|  | XP\_065069500\_15627-model5.56 | -9.32553 |
|  | XP\_065069500\_15627-model5.57 | -9.32553 |
|  | XP\_065069500\_15627-model5.58 | -9.32501 |
|  | XP\_065069500\_15627-model5.59 | -9.3231 |
|  | XP\_065069500\_15627-model5.60 | -9.32178 |
|  | XP\_065069500\_15627-model5.61 | -9.30922 |
|  | XP\_065069500\_15627-model5.62 | -9.30063 |
|  | XP\_065069500\_15627-model5.63 | -9.30063 |
|  | XP\_065069500\_15627-model5.64 | -9.30063 |
|  | XP\_065069500\_15627-model5.65 | -9.28235 |
|  | XP\_065069500\_15627-model5.66 | -9.28235 |
|  | XP\_065069500\_15627-model5.67 | -9.28164 |
|  | XP\_065069500\_15627-model5.68 | -9.28102 |
|  | XP\_065069500\_15627-model5.69 | -9.28047 |
|  | XP\_065069500\_15627-model5.70 | -9.27507 |
|  | XP\_065069500\_15627-model5.71 | -9.26816 |
|  | XP\_065069500\_15627-model5.72 | -9.23464 |
|  | XP\_065069500\_15627-model5.73 | -9.22625 |
|  |  |  |
